# Continuous Medicaid coverage: a policy strategy to improve access to care for high-risk neonates

**DOI:** 10.1038/s41372-025-02554-0

**Published:** 2026-03-18

**Authors:** Shetal Shah, Heather L. Brumberg

**Affiliations:** Division of Neonatology, Department of Pediatrics, Maria Fareri Children’s Hospital, New York Medical College, Valhalla, NY USA

**Keywords:** Health policy, Health care economics

## Abstract

Health insurance coverage is a determinant of access to care, particularly for high-risk patients or those with chronic diseases. Medicaid is the largest insurer of children and state-based differences in eligibility requirements often lead to lapses in coverage in early childhood. In recent months, several states have initiated continuous coverage provisions with Medicaid, allowing eligible children to be insured until ages 3-5 years without the need for re-enrollment. We review data on the association of insurance coverage with health outcomes for children and suggest continuous coverage policies may benefit medically-complex graduates of neonatal intensive care units (NICU), highlighting the need for neonatologists to be vocal advocates for states to adopt this pattern of coverage.

## The health insurance coverage landscape for children

Nationally 92% of the US population has health insurance, which is associated with improved health outcomes [1, 2]. Compared to those without health insurance, insured adults are more likely to have a usual source of healthcare (primary care physician) and exhibit lower rates of cardiovascular and all-cause mortality [3]. Similarly, young adults, children and low-income adults with health coverage are 2–3 times more likely to have received preventive health services and demonstrate lower rates of non-emergent emergency room visits [4–7]. For families, another key benefit is health insurance mitigates the financial burden of care [1].

Unique among industrialized countries, children’s health coverage in the United States (US) is provided by a patchwork of commercial, self-pay and public sources, including programs such as TriCare, Medicaid, Medicaid Managed Care Plans, commercial employer-sponsored coverage and the Children’s Health Insurance Plan (CHIP) [8–11]. Roughly 36.4 million children, or 49% of the pediatric population, are covered by commercial insurers via employee-sponsored programs [12]. The landscape of public coverage for children is dominated by Medicaid, which in addition to CHIP, covers 30.6 million (41%) of the pediatric population [12]. Among children covered by Medicaid, 50% have special health needs [13]. The program is the single largest payor for newborns, insuring 40% of all US births, but even higher percentages of early term (45%), late preterm (48%) and early preterm (50%) births [13, 14]. Further, Medicaid-insured newborns are disproportionately from minoritized populations, including 60–65% of Non-Hispanic Black and Hispanic births [9, 14, 15].

A federal-state partnership in which states retain partial authority in benefits covered, eligibility and payment rates, the current Medicaid program is disordered, mis-matched and inconsistent. Since individual states hold dominion over many aspects of their respective Medicaid programs, large regional variability exists among state programs, specifically related to income eligibility thresholds, payment levels and duration of coverage prior to re-enrollment. Nationally, the median income eligibility threshold for Medicaid across states was 195% of the Federal Poverty Level (FPL) for children under a year of age, but ranged from 142% in Louisiana (and Illinois) to 380% of the FPL in Iowa [16]. For older children up to 5 years, the median income parental income eligibility level is 148% of the FPL, but ranges from 138% in Maryland (and Oregon) to 324% in the District of Columbia [16]. As a result of these differences in income eligibility, uninsurance rates vary greatly across states. For example, roughly 1 in 5 residents of Texas (17.7%) lack health coverage, compared to only 2.8% in Massachusetts [3].

Federal policies influence the rate of uninsured children. The 2010 Affordable Care Act incentivized states to expand Medicaid for all individuals and families up to 138% of the federal poverty level, which facilitated a reduction in the children health uninsurance rate from ~10% to 4.7% in 2015 [9, 10, 17]. However, a year later, the number of uninsured children increased by 677,900 (~1%) [18, 19]. This increase resulted from several factors, including the de-funding of outreach programs meant to promote Medicaid and assist families with enrollment. Another related cause of this increase was federal approval of Medicaid waivers, which allowed states to change aspects of coverage such as creating more complex re-enrollment applications or attaching parental drug testing or work requirements to Medicaid coverage, both of which dis-incentivize parents to enroll children [18, 19].

Beyond uninsurance, it is also important to evaluate underinsurance, defined as having health insurance coverage insufficient to meet health needs without added financial burden, often resulting in the forgoing of care. Medicaid protects access to care not only for low-income newborns with chronic health needs, but plays a role in supporting families at higher income levels who may be underinsured on their employee-sponsored insurance plan. For the underinsured, Medicaid may support durable medical equipment, home health care, medical supplies and certain off-label medications—services not provided by high-deductible plans which may prove initially appealing to families due to their low premiums [14]. Further, Medicaid’s role as a secondary insurer is important for those insurance plans with annual spending limits. This role as a secondary insurer is especially relevant to the extremely low birthweight population, as prolonged NICU stays frequently eclipse annual limit thresholds [20].

## Health needs of the preterm infant require long terms of coverage and access to care

The complex medical needs of preterm infants make them vulnerable to procedural lapses in Medicaid coverage, which occur when infants remain eligible for Medicaid based on income, but are removed from the program because they did not complete re-enrollment procedures or formally re-verify their income. These procedural losses in coverage render them uninsured and may disconnect these chronically-ill patients from care. Preterm birth and comorbidities of prematurity incur higher rates of health utilization, increasing cost and requiring significant medical resources [20]. Although rates of the comorbidities of prematurity have decreased over time with improvements in care, high-grade intraventricular hemorrhage (~15%), periventricular leukomalacia (~5%), necrotizing enterocolitis (~8%) and retinopathy of prematurity (>3) (~12%) rates remain sizeable [21]. Bronchopulmonary dysplasia now occurs in roughly ½ of premature babies born below 29 weeks who survive, a consequence of increased survival and improvements in maternal and neonatal care [22].

The need for ongoing care continues post NICU discharge. Infants ≤32 weeks average 20 pediatric outpatient visits per year, most of which are not standardized well-child visits—and require a mean of 6 prescriptions per year [23]. For infants below 26 weeks gestation, these visits increased to 29 per year with a need for roughly 9 prescriptions [23]. Beyond the index hospitalization, extremely preterm babies average 1 rehospitalization and over 2 Emergency Room visits in the first year of life, with rates only slightly lower among preterm infants of older gestational age [24]. These visits are substantially higher than the 6 well-child visits recommended in the first year of life for otherwise healthy, full-term newborns [25]

Currently, federal Medicaid rules require 1 year of uninterrupted coverage from birth in all states [26]. However, for children with chronic conditions, studies demonstrate only 1 year of continuous Medicaid coverage may not be sufficient to optimize health outcomes. While this data is derived mainly from older pediatric patients, it suggests high-risk infants may also benefit from longer durations of uninterrupted coverage. For example, the American Academy of Pediatrics recommends testing and ongoing developmental surveillance in primary care for former preterm infant through 5 years of age, which could facilitate longer term subspecialty referral or specific developmental testing. This suggests continuous Medicaid coverage through early childhood may better suit the needs of high-risk infants upon NICU discharge [27].

## Past Medicaid policies which prolong coverage increase the number of insured children

The Medicaid program has previously experimented with providing longer periods of coverage for eligible children. These past policies serve as laboratories for examining the impact of longer coverage duration on the number of uninsured children. In 2020, federal legislation provided a temporary increase in payments to states if they maintained continuous Medicaid coverage through the duration of the COVID public health emergency [10]. This meant state governments would receive more federal matching funds to support their state’s Medicaid program if they agreed to cover children through the pandemic without requiring families to periodically re-enroll by re-demonstrating income levels and eligibility. Re-enrollment processes, created by states are often complex, and certain states have used the process to create artificial barriers to providing Medicaid. For example, several states offer applications in a limited number of languages and provide technical assistance via a phone-system which is difficult to navigate for those with limited English proficiency. These barriers result in fewer re-enrollment applications and decreased state expenses [28]. The landmark provision to extend Medicaid during the federal public health emergency without the need to formally re-enroll was associated with a reduction in the child uninsurance rate to <5% from 2020–22 [12, 29]. This pandemic-related continuous Medicaid policy moved over 7 million children into Medicaid during those 3 uninterrupted years. The majority of these children obtained coverage after parental loss of employee-sponsored coverage, which was lost due to increases in unemployment during the pandemic [29].

Several states used the prolonged duration of continuous coverage as a roadmap to extend periods of Medicaid from birth through early childhood—a benefit which may seismically assist NICU graduates with accessing services to meet ongoing special healthcare needs. However, at the end of the public health emergency in the Spring of 2023, states disenrolled children and families, the majority for failing to demonstrate continued income eligibility [10]. Therefore, by April of 2024, Medicaid reported losses in coverage for ~4.6 million children, nearly ½ of whom gained coverage during the pandemic [30].

Currently, 8 states, Oregon, Washington, New Mexico, New York, Colorado, Hawaii, Minnesota and Pennsylvania are transitioning toward uninterrupted Medicaid coverage through 6 years of age, a timepoint where a majority of immunizations and early preventive care are completed and some developmental aspects of care are occurring within school systems [20, 31] (Table 1). Similar measures are in development in Maryland, Ohio, and California [20, 31]. These measures are a direct result of the experience with continuous coverage children experienced during the COVID pandemic.

**Table 1:** Summary of Medicaid Continuous Coverage Policies in Selected States.

State	Summary
Colorado	Medicaid continuous eligibility until 3 yrs. [48, 49]
Washington State	
New Mexico	Medicaid coverage through 6 yrs. [50–52]
New York	
Minnesota	Medicaid coverage through 6 yrs, with 12 months of continuous eligibility to age 19 yrs. [53]
Pennsylvania	Medicaid coverage through 6 yrs., with 12 months of continuous eligibility to age 19 yrs for high-risk children. [54]
Hawaii	
Oregon	Medicaid coverage through 6 years (yrs.) of age, with 24 months of continuous enrollment for children >6 yrs. [55, 56]

Currently, 8 states have initiated or approved provisions to enroll eligible children in Medicaid for greater than 1 year.

## Impact of longer durations of Medicaid coverage on children’s health outcomes

For children with chronic diagnoses, lengthening the period of public coverage yields improved access to care and is associated with better health outcomes [20]. Children with diabetes without gaps in coverage are more likely to have seen an endocrinologist [32]. Uninsurance or partial-year coverage was also associated with roughly 5x higher odds of being unable to afford asthma medication in children [33]. For children with special health needs, longer periods of Medicaid coverage increase odds of accessing preventive and specialty care [34]. Even among children who cycle between public and commercial insurance, fewer lapses in coverage is associated with a three-fold lower odds of having an unmet health need [34, 35]. (Table 2)

**Table 2:** Summary of Health Outcomes Associated with Longer Durations of Coverage in Medicaid for Selected Populations.

Population	Associated Health Outcomes
Birth to 1 year of age	• Savings of $292–696 Million [31] • Increased preventive care visits [43, 57] • Increased up-to-date immunization status [43]
Children under 18 yrs.	• Increased likelihood of seeing a specialist among children with a chronic disease [32] • Increased medication access [33] • Lower risk of having unmet health needs [34] • Annual savings of ~$10.1 Billion [37]
Postpartum Women	• Increased access to family planning services [40] • Increased smoking cessation treatment [41] • Increased anti-hypertension treatment and lipid screening among high-risk mothers [41, 42] • Improved post-delivery glucose monitoring for mothers with gestation diabetes [41, 42]

Longer durations of continuous enrollment in health insurance coverage is associated with cost savings, healthcare access to primary and subspecialty care and selected improved health outcomes in postpartum women.

Vasan et al. found states that newly adopted continuous coverage policies saw a 4.6% increase in Medicaid enrollment [36]. Studying a Texas-based cohort of Medicaid-eligible children, those continuously insured experienced greater healthcare access and fewer out-of-pocket costs [37]. Longer periods of uninterrupted healthcare coverage demonstrate long-term cost-effectiveness when large pediatric populations are insured. Though a seven-state analysis of providing 1 year of pediatric continuous Medicaid eligibility incurred a modest 2.2% increase in state costs, overall, state Medicaid spending may be reduced with a longer period of coverage and with a greater number of patients insured [38]. Extrapolating from this data, longer coverage periods and prevention of uninsurance result in an estimated $2,886 per patient savings, and an overall annual savings of $10.1 Billion dollars to healthcare systems [37].

Continuous coverage policies that extend Medicaid coverage beyond a year from birth without re-enrollment requirements may disproportionately benefit under-represented children. Minoritized children are both more likely to undergo prolonged periods of uninsurance and are over-represented in Medicaid [14]. Black children are particularly vulnerable, as even compared to Hispanic children, they are at 3 times higher risk for being uninsured for at least a year [14]. Although not often articulated in advocacy efforts, continuous coverage can be considered an important strategy to address health inequity and disparities in access [14].

## The trend of extending Medicaid coverage continuously without re-enrollment

### Post-partum women

Actions by states to extend continuous coverage from birth through 6 years build on a recent trend by states to extend durations of coverage for high-risk populations. For example, as a response to high levels of maternal mortality and racial/ethnic disparities in post-partum deaths among Non-Hispanic Black and American Indian/Alaskan Native, the 2021 American Rescue Plan conferred an option for states to amend Medicaid programs to extend post-partum coverage to 12 months (10 months beyond the federally-required minimum of 60 days) [39, 40]. Incentivized by a federal contribution to the increased costs of providing this coverage extension, 32 states quickly elected to prolong post-partum coverage in 2023. Currently, 49 states have implemented this provision – addressing family planning services, smoking cessation and depression screening for women after birth [40, 41]. The postpartum extension of Medicaid was also associated with improved outcomes for mothers with chronic disease. Mothers with hypertension or preeclampsia were more likely to receive lipid screening and anti-hypertensive treatment if insured [41, 42]. Similarly, among women with gestational diabetes, improved post-partum glucose monitoring is seen under prolonged periods of Medicaid coverage after birth [41, 42].

### Extending coverage from birth to 1 year

In another example, the 2023 Consolidated Appropriations Act mandated all states provide a full year of uninterrupted coverage to children in Medicaid and CHIP. The requirement targeted the first year of life because of its role in identifying developmental delays and in administering preventive health services. Overall, the economics of extending this policy are favorable. Public insurance coverage for the first 12 months of life collectively saved families an estimated $292 million dollars in healthcare savings – stemming from reduced physician, emergency room and prescription costs. However, the federal and state governments did spend $696 million in additional Medicaid costs to provide this longer duration of coverage to babies. However In the context of government budgets, the relative increase in federal and state spending is minimal, especially in the context of family savings and the demonstrated health benefits of this policy [31, 43].

Coverage for a full year after birth is also associated with improved immunization status [43]. Finally, the Centers for Medicare and Medicaid Services released rules limiting the duration of Short-Term, Limited Duration Insurance Plans, which are exempt from Affordable Care Act’s mandated health plan requirements to cover certain procedures and doctor visits. Absent the need to adhere to minimum standards, these short-term plans are often of poor quality [44]. Initially meant to cover patients through a predetermined space in coverage (such as between employee-sponsored coverage which switching jobs), these plans were often renewed by participants attracted to their lower price/premiums. However, in renewing these plans, these families were not subject to the mandates 1 year of coverage and other ACA-mandated protections [39]. Curtailing use of these short term plans is expected to drive more families into both public and commercial insurance plans with longer coverage durations prior to renewal.

### Advocacy by neonatologists

As more states evaluate the potential benefits of continuously covering children in Medicaid through school age, cooperation among those who care for pregnant mothers, newborns and young children is essential [45]. Policies which extend the duration of coverage have the ability to ensure NICU-graduates with comorbid conditions have facilitated access to subspecialists throughout early childhood, potentially optimizing their care and improving long-term outcomes at school entry. Neonatologists are natural advocates for newborns. Promoting enduring health insurance coverage for high-risk infants and mothers requires direct and active engagement with policy makers. Given the disproportionate benefits to our specific patient population, state actions on continuous Medicaid coverage provide a natural opportunity for collaborative advocacy between neonatologists and pediatricians who take over the care of these infants after NICU discharge. NICU specialists can educate lawmakers about the complex long-term, health challenges faced by neonatal patients and give voice to the ongoing hurdles families face accessing care. Pediatricians can detail the obstacles they encounter assisting families with re-enrolling in Medicaid once a medically-complex patient is dis-enrolled and can elaborate on the impact of providing this uncompensated assistance on practice finances.

While disheartening, the passage of the austere Medicaid cuts contained within the “One Big Beautiful Bill” Budget Reconciliation legislation should not deter advocacy efforts toward continuous coverage [46]. The need to reduce state Medicaid spending to meet reduced federal contributions is re-energizing discussions about how to streamline Medicaid to increase efficiency and consequently, reduce long-term cost over the 10 year span of the legislation. For example, while continuous coverage is estimated to increase short-term costs by $112 million, savings associated with the longer durations of coverage outlined above suggest net cost savings over a decade [46]. Neonatologists are ideal messengers of the long-term value associated with this Medicaid policy, especially as greater cost-savings are realized in patients with comorbidities. Specific neonatal actions may include: engaging with advocacy organizations such as the local American Academy of Pediatrics Chapter or state Medicaid office, meeting with legislators with oversight of health policy to educate them on the importance and cost-savings of extending early childhood coverage, drafting opinion/editorial pieces and testifying during state budget discussions in support of this investment [47].

Extending the duration of Medicaid coverage from birth through early childhood is a provision gaining traction across state legislatures. However, without neonatologist input, those patients who stand to benefit the most from this policy may be marginalized and left out of the discussion.
